# Tick-Borne Encephalitis—Review of the Current Status

**DOI:** 10.3390/jcm12206603

**Published:** 2023-10-18

**Authors:** Malgorzata Kwasnik, Jerzy Rola, Wojciech Rozek

**Affiliations:** Department of Virology, National Veterinary Research Institute, Al. Partyzantow 57, 24-100 Pulawy, Poland; jrola@piwet.pulawy.pl (J.R.); wojciech.rozek@piwet.pulawy.pl (W.R.)

**Keywords:** tick-borne encephalitis, TBEV, arbovirus, Flaviviridae, Ixodes, vector-borne

## Abstract

The tick-borne encephalitis virus (TBEV) is the arboviral etiological agent of tick-borne encephalitis (TBE), considered to be one of the most important tick-borne viral diseases in Europe and Asia. In recent years, an increase in the incidence of TBE as well as an increasing geographical range of the disease have been noted. Despite the COVID-19 pandemic and the imposition of restrictions that it necessitated, the incidence of TBE is rising in more than half of the European countries analyzed in recent studies. The virus is transmitted between ticks, animals, and humans. It seems that ticks and small mammals play a role in maintaining TBEV in nature. The disease can also affect dogs, horses, cattle, and small ruminants. Humans are incidental hosts, infected through the bite of an infected tick or by the alimentary route, through the consumption of unpasteurized milk or milk products from TBEV-infected animals. TBEV infections in humans may be asymptomatic, but the symptoms can range from mild flu-like to severe neurological. In Europe, cases of TBE are reported every year. While there is currently no effective treatment for TBE, immunization and protection against tick bites are critical in preventing this disease.

## 1. Introduction

Tick-borne encephalitis (TBE) is an arboviral disease caused by the TBE virus (TBEV), a member of the Flaviviridae family, as are the etiological agents of dengue fever, yellow fever, and Japanese encephalitis. Tick-borne encephalitis is a serious health problem in Europe and Asia [1,2]. In recent years, a rise in the incidence of TBE as well as an expansion of the geographical range of the disease have been evident. In 2020, 24 European Union/European Economic Area (EU/EEA) member countries reported 3817 cases of TBE [3]. The virus is transmitted between ticks, animals, and humans, so it can be considered in the context of a one health perspective [4]. Humans are incidental and dead-end hosts infected mainly through the bites of hard ticks. Another route of TBEV transmission may be the ingestion of unpasteurized milk or dairy products from infected animals [5]. In this article, we discuss the characteristics of TBEV, its structure, and the phylogenetic relationship of the circulating strains. We describe the reservoir, vectors, and transmission routes of the virus; its geographic range; and the clinical symptoms and diagnostics. We discuss the potential impact of the COVID-19 pandemic and climate change on the incidence of TBE and the available prevention and treatment methods.

## 2. Virus Structure

**Figure 1:**
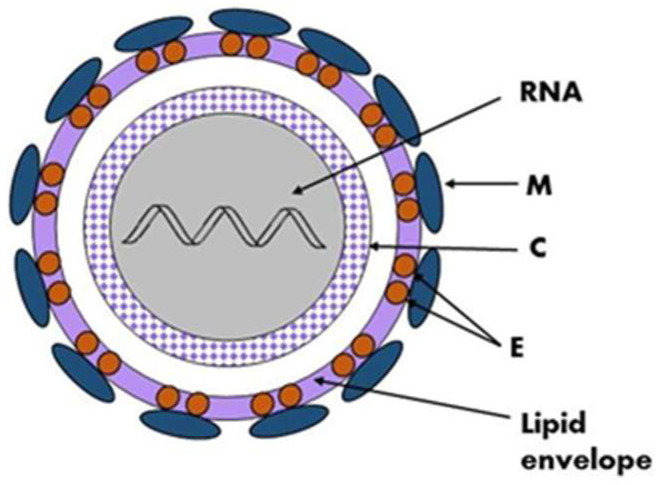
Schematic structure of the TBEV virion, the viral genome (RNA) and the C protein form a nucleocapsid surrounded by a lipid envelope in which glycoproteins E and M are embedded.

The tick-borne encephalitis virus is spherical or quasi-spherical, lipid-enveloped, and approximately 50 nm in diameter, and it contains a positive, single-stranded RNA genome that acts as mRNA for translation. Although the approximate size and shape of TBEV and other flaviviruses are estimated, there are variations in size due to factors such as the genetic diversity in the virus population, changes during the maturation process, and the methods used for the imaging and analysis of virions [6]. The virion is composed of three structural proteins: the envelope (E), membrane (M), and capsid (C) proteins (Figure 1). Seven non-structural (NS) proteins—NS1, NS2A, NS2B, NS3, NS4A, NS4B, and NS5—have also been identified in infected cells. Non-structural proteins play an important role in virus replication, the processing of the structural proteins, and the modulation of host cell function. The M glycoprotein is primarily synthesized as a precursor (prM) that interacts with glycoprotein E and protects its fusion loop from premature activation [7,8]. The nucleocapsid consists of the genome and the C protein and is surrounded by the viral envelope, which consists of both M and E glycoproteins and host-cell-derived lipids. Glycoprotein E is the major antigen of TBEV and is responsible for receptor binding and membrane fusion [8,9]. The glycoprotein-E-coding gene is commonly sequenced and analyzed, but a pairwise distance analysis indicated that it has evolutionary patterns distinct from other TBEV genomic regions [10].

In recent years, it has been confirmed that the structure and sequence of non-coding RNA regions of Flavivirus genomes are of great functional importance. The 5′ untranslated region (UTR) and 3′ UTR of TBEV are postulated to be important for genome replication. These noncoding fragments dimerize, leading to the cyclization of the genome via the formation of a panhandle structure [11,12,13]. Hirano et al. reported that a cis-acting RNA element was identified in the 5′ UTR of TBEV that mediates neurovirulence by hijacking the host mRNA transport system, thus allowing the transport of TBEV genomic RNA to neuronal dendrites, where it replicates locally. Neuronal granules are involved in the transport of TBEV genomic RNA, and the hijacking of their system for the transport of viral genomic RNA in dendrites demonstrated in the results of Hirano et al. indicates the neuropathogenicity of the virus and explains how the viral infection can result in severe neurological diseases [14]. A recent study investigated the role of the predicted secondary RNA elements of the first 107 nucleotides of the genome (stem-loop A, SLA) [11]. Mutations within individual SLA structures (Core 0, Stem 1, Stem 2) affected virus replication, infectivity, and spread, but an effect on viral translation was not suggested.

## 3. Phylogenetic Analysis of Circulating Virus Subtypes

**Figure 2:**
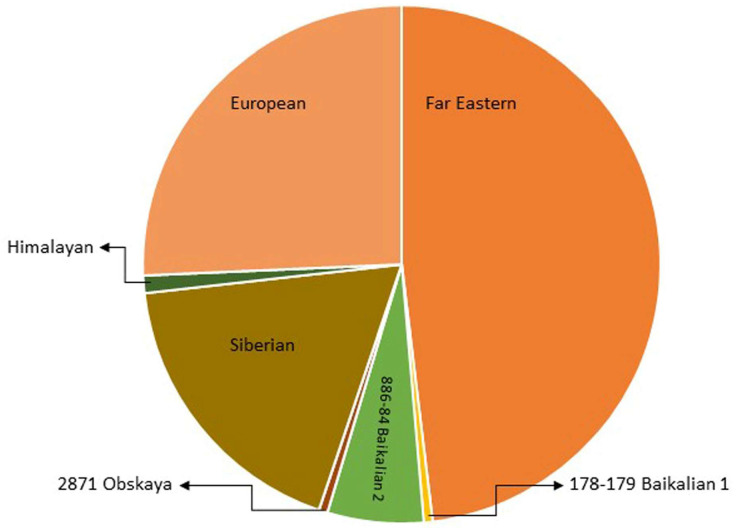
The pie chart shows the abundance of identified TBEV strains within subtypes. 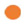 Far Eastern group: 88 TBEV strains; 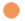 European: 47 strains; 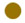 Siberian: 33 strains; 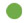 886-84 Baikalian 2: 11 strains; 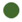 Himalayan: 2 strains; 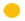 178-179 Baikalian 1: 1 strain and 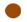 Obskaya 2971: 1 strain [10,16,19,24].

Traditionally, TBEVs have been classified into three subtypes: the European (TBEV-Eu), the Siberian (TBEV-Sib), and the Far-Eastern (TBEV-FE). Recently, Baikalian (TBEV-Bkl) and Himalayan (TBEV-Him) subtypes have been distinguished [15,16,17]. The assumption that if the open reading frame nucleotide sequence of two viruses differs by less than 10%, the two viruses belong to the same subtype guided the recent analyses, and they pointed to seven subtypes of TBEV: TBEV-Eu, TBEV-Sib, TBEV-FE, TBEV-Ob (TBEV-2871), TBEV-Him, TBEV-Bkl-1 (178-79), and TBEV-Bkl-2 (886-84) (Figure 2) [10]. Another phylogenetic analysis using the Nextstrain framework and based on more than 220 complete TBEV genomes proposed TBEV-Bkl1, TBEV-Bkl-2, and TBEV-Him as separate clades in addition to the three major subtypes TBEV-Eu, TBEV-Sib, and TBEV-FE [18].

The viral strains within the three main subtypes of TBEV, TBEV-Eu, TBEV-Sib, and TBEV-FE are believed to be descended from a common ancestor and have evolved independently. Recent studies on TBEV strains isolated near Lake Baikal in Russia, TBEV-Bkl-2 (886-84), have shown that these strains have a mosaic genome: some parts are more closely related to viruses from the Siberian group, while others are more closely related to the Far-Eastern group. Therefore, the Baikalian subtype of TBEV is postulated as evident of recombination between the Siberian and Far-Eastern subtypes [19].

The phylogenetic groups of TBEV may differ in their clinical presentation. For the European subtype, the fatality rate has been estimated at below 2% [20]. The disease caused by European subtypes of TBEV is usually biphasic with a viremic phase associated with a fever and myalgia, and in some patients, is followed by neurological symptoms of varying severity [1]. The Far-Eastern subtype is considered the most pathogenic, with a mortality rate estimated at up to 40% by some authors [21,22]. The Siberian subtype typically results in a less severe disease than that caused by the TBEV-FE subtype, with a fatality rate of 6–8%, and it may be associated with chronic TBE [23]. The TBEV-Ob (2871) strain was isolated from *Ixodes pavlovskyi*, and it has not been detected in humans, so its pathogenicity to humans is unknown. The virulence of the strain has been confirmed in laboratory mice. According to the classification based on the invasiveness index, the TBEV-Ob strain belongs to the group of the most common strains from Western Siberia [24]. Him-TBEV was detected in a wild rodent, *Marmota himalayana*, at the Qinghai–Tibet Plateau in China. An analysis of 17 amino acid residues associated with the pathogenicity of TBEV showed that Him-TBEV shares nine substitutions that are specific to pathogenic strains and five substitutions that are specific to strains isolated from subclinical cases. The pathogenic-associated amino acid substitution profile of the Him-TBEV strain is similar to the low-pathogenic TBEV Oshima strain [16]. The ability of TBEV-Bkl-2 (886-84) to cause lethal focal forms of encephalitis, as well as the results of laboratory tests, indicate the high pathogenic potential of this group [25]. However, assigning a certain level of pathogenicity to strains of a given subtype may be misleading. Some studies have shown that different strains within certain TBEV subtypes may show a variable virulence [26,27].

## 4. TBEV Reservoirs, Vectors, and Transmission

Hard ticks of the family Ixodidae act both as vectors and reservoirs of TBEV. *Ixodes ricinus* occurs especially in central, northern, and eastern Europe, and *I. persulcatus* is found in parts of the Baltic States, Finland, Russia, and Siberia [28]. Field studies and experimental findings indicate that other species of ticks might also be effective TBEV vectors. Natural infections with TBEV were reported in 16 species of ixodid ticks more than 30 years ago [6]. Currently, at least eight species are known to be able to transmit the TBE virus, and so far, the virus has been isolated from at least 14 other species [29]. In Central Europe, TBEV has been revealed in some species of “hard” ticks: *Ixodes persulcatus*, *Ixodes ricinus*, *Ixodes hexagonus* [30], *Ixodes arboricola* [31], *Haemaphysalis punctate* [32], *Haemaphysalis concinna* [33], *Dermacentor marginatus*, and *Dermacentor reticulatus* [34]. *Ixodes gibbosus* is considered a marginal vector in the Mediterranean region [32]. Nosek et al. experimentally proved the vector competence of *Haemaphysalis inermis* for TBEV [35]. 

**Figure 3:**
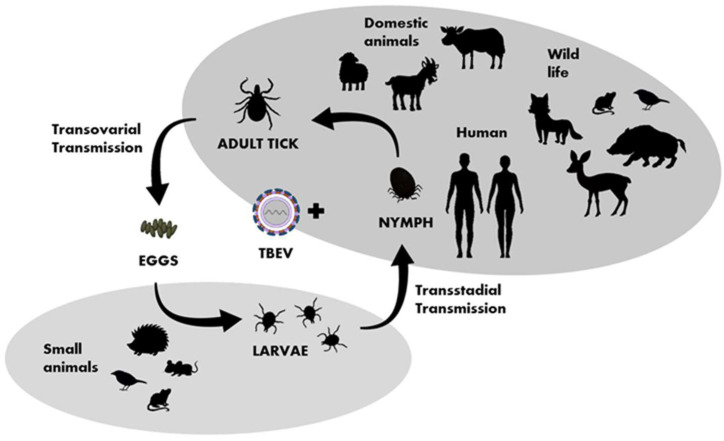
Transmission of TBEV in the life cycle of ixodid ticks, shaded fields indicate the host group characteristic for particular developmental stages of ticks.

TBEV circulates in small, geographically defined areas, so-called “natural foci”. This cycle involves ticks as vectors and small rodents, insectivores, birds, and large mammals as hosts (Figure 3). Ticks can become infected by feeding on viremic hosts (viremic transmission) or by co-feeding with an infected tick (non-viremic transmission) [36]. Other ways of transmitting the virus include vertical transovarial transmission (via eggs laid by an infected female) and transstadial transmission (between developmental stages of ticks). Horizontal sexual transmission may take place between both ticks and warm-blooded hosts [37]. The transstadial and transovarial transmission of TBEV co-exist and take place simultaneously along with sexual, non-viremic, and other transmission modes.

The important hosts and reservoirs are small mammals such as rodents (mice and voles), insectivores (hedgehogs and moles), and carnivores (foxes). Rodents are amplifying, asymptomatic hosts of the virus. Prolonged viremia has been confirmed in some rodent species, such as the bank vole (*Clethrionomys glareolus*). Thus, ticks and small mammals can play a key role in maintaining TBEV in an environment. It is suspected that birds also play a role in the virus’ spread [38,39]. A phylogenetic analysis after the introduction of TBEV to the United Kingdom [40] and studies conducted in Finland and Japan [41,42] indicate the involvement of migrating birds in the spread of the virus. Ticks are believed to have different specific ranges of target animals at different life stages, e.g., adult ticks attack mainly large animals, while nymphs and larvae attach to small- and medium-sized animals, including birds [43,44]. In Europe, larger animals, mainly wild deer such as roe deer (*Capreolus capreolus*), are important hosts for adult ticks [45]. In Poland, seroprevalence against TBEV was recorded in European bison (*Bison bonasus*), the largest European herbivore, at a level of 63.5% [46]. Domestic animals may also be infected with TBEV, with goats in particular being commonly infected with TBEV because of their grazing pattern and preference for scrub. Infections with TBEV in sensitive domestic animal species such as dogs, horses, and animals kept in captivity, e.g., macaques, can be severe and may manifest with clinical signs similar to those seen in severe human cases [47]. In contrast, TBE is typically asymptomatic in domestic ruminants; however, there are rare descriptions of symptomatic disease [48]. The main route of TBEV transmission for humans is tick bites, or less often, alimentary, when the consumption of raw milk or milk products from infected ruminants introduces the virus [39,49,50]. The tick-borne encephalitis virus is relatively sensitive to temperatures and detergents, but remains infectious in gastric juice (pH of 1.49–1.80) for up to two hours [51]. It is estimated that approximately 1% of all TBEV infections in humans are transmitted by the alimentary route [52], and such infections have been reported in at least 10 European countries, with the highest numbers in Slovakia, Hungary, Czechia, and Poland. In Croatia, Germany, and Slovenia, single cases of TBE infections by the alimentary route have been reported [5].

Single cases of TBE transmitted by other routes have also been reported: aerosol infections among laboratory personnel [53], blood transfusions [54], and organ transplantation [55]. Transmission from an infected mother to her baby through breast milk is also suspected [5,56]. An example of a fatal TBEV infection following organ transplantation involved three patients who received organs from a single donor (two received a kidney and one received a liver). All the recipients developed encephalitis 17–49 days after the transplantation, which led to death. The presence of TBEV was confirmed using RT-PCR in the recipients and the donor, and sequencing confirmed the presence of the same virus strain. In this case, the course of the TBEV infection may have been complicated by pharmacological immunosuppression. It would be advisable for organ donors to be screened for TBEV, especially if they come from endemic regions [55].

## 5. The Geographical Range and Frequency of Infections

It is accepted that TBE is endemic in several European countries, mainly in northern, eastern, and central Europe [57]. According to a report by the European Centre for Disease Prevention and Control (ECDC), the most affected EU/EEA countries in 2020 included Lithuania (24.3 TBE cases per 100,000 individuals), Slovenia (8.9), Czechia (7.9), Latvia (7.8), Estonia (5.3), Slovakia (3.4), Austria (2.8), Sweden (2.6), and Finland (1.6). The highest numbers of confirmed cases were reported by Czechia (849), Germany (705), and Lithuania (679). Germany’s confirmed case number reflects its large population, and the rate in the country per 100,000 individuals was <1. In other countries, the incidence was relatively low (the estimated per-100,000 rate was also <1 in Belgium, Bulgaria, Croatia, France, Hungary, Italy, Norway, and Poland), or no cases were reported (Cyprus, Denmark, Iceland, Liechtenstein, Malta, Portugal, and the UK). According to the data received by the ECDC, the notification rate for TBE remained stable at 0.6 from 2016 to 2018, after which it increased to 0.7 in 2019 and 0.9 in 2020 [3].

In Asia, the virus is widespread in Russia, Kazakhstan, Kyrgyzstan, Mongolia, China, Korea, and Japan [58]. Chen et al. (2019) reported that the annual incidence of TBE in China increased from 0.09 to 0.44 per 100,000 individuals between 2007 and 2018. The cases of TBE were mainly distributed in northeast China, in DaXingAnLing Prefecture and ChangBaiShan [59]. In Japan, four cases of TBE were reported in a wide area of Hokkaido between 2016 and 2018, and were the only cases since 1993. In epizootiological surveys in Japan, the seropositivity for TBEV was estimated at approximately 10% to 20% in wild rodents in southern Hokkaido [58]. Seropositive dogs, horses, and deer were also sporadically detected [60,61]. Although cases of human encephalitis are increasing in the Republic of Korea, no cases of TBE have been confirmed so far. In 2008, TBEV was isolated from wild *Apodemus agrarius* rodents in Hapcheon, Gyeongsangnam-do [62].

The emergence of TBEV is being observed in new territories. The first detection of TBEV in the UK was confirmed in ticks collected in the Thetford Forest area of East Anglia in 2018. The second detection of TBEV in the UK was in ticks on the border of Hampshire and Dorset in southern England. A sequence analysis indicated that the TBEV-UK-Thetford strain was similar to the Norwegian Mandal 2009 strain. The second TBEV isolate was closely homologous to a strain identified in the Netherlands [63]. In the Netherlands, the serological screening of roe deer showed TBEV antibodies with a prevalence of 2%. Additionally, TBEV RNA was detected in two ticks collected in the same region of the Netherlands. The analyzed sequences from both ticks grouped within the TBEV-Eu subtype complex were, in fact, identical, and were designated as TBEV-NL. Subsequent studies confirmed TBE infections in two human cases [64]. Holzmann et al. reported the transmission of TBEV in 2008 via unpasteurized goat milk to six humans and four domestic pigs in an Alpine region in Austria [49]. This is an example of the extension of the TBEV range to the mountainous regions of Austria, above altitudes previously considered virus-free. 

## 6. Clinical Symptoms and Diagnosis

The incubation period of TBE ranges from 4 to 28 days [20]. The incubation after a foodborne infection is usually shorter, and up to 4 days [1]. Some studies have suggested a correlation between the length of the 3′ UTR of TBEV and the incubation period of the disease. In the case of viral strains with a 3′UTR sequence shorter than 200 nucleotides, the incubation period for suckling mice was longer than 5 days [13]. Other factors that may impact the incubation period are the viral load and subtype, the host’s innate and specific immunity, and flavivirus resistance gene structures [6]. The course of a TBEV infection varies and depends on the age and immune status of the infected person and the characteristics of the particular TBEV strain. Generally, an infection with TBEV can be symptomatic or asymptomatic. A symptomatic infection can be monophasic (with or without neurological symptoms) or biphasic, as it is in most patients. In the first stage of the biphasic course, nonspecific symptoms occur, such as a fever, headaches, and muscle pain lasting up to one week, and 70% of patients are diagnosed with leukopenia and thrombocytopenia. In the second phase, there are symptoms of encephalitis in the form of a persistent fever, headaches, insomnia, confusion, possible vomiting, a stiff neck, muscle pain, and paresis [1]. The second phase of the disease can also manifest as hemorrhagic syndrome [65]. In this phase of the disease, an increase in the white blood cell count, an elevated C-reactive protein (CRP) level, and a higher erythrocyte sedimentation rate (ESR) can be observed [66].

Tick-borne encephalitis is usually diagnosed clinically and serologically in the neurological phase of the disease [67,68]. Enzyme-linked immuno-sorbent assays are the current method of choice for the rapid detection of TBE-specific IgM and IgG antibodies in the sera of unvaccinated patients. However, IgM antibodies are not detected in serum or CSF in the early phase of the disease [69,70]. Specific IgM antibodies are usually detected in the serum when neurological symptoms occur, and the IgM response in CSF occurs later than it does in serum [71]. A study by Reusken et al. demonstrated the importance of IgM determination in serum and cerebrospinal fluid to diagnose a TBEV infection. An analysis of ELISA results showed a lack of IgG specificity. Additionally, the CSF/serum IgG antibody index can support a diagnosis in cases of chronic disease or when IgM has disappeared [72]. 

The use of molecular diagnostic methods, such as a TBE-specific PCR, allows for the identification of all TBEV subtypes in the early phase of the disease [73,74]. The molecular technique is of lesser importance in healthcare practice, since a diagnosis is usually required for patients with neurological symptoms of the disease. Viral RNA can be detected in blood or serum during the first phase of the infection, when the patients are asymptomatic or the symptoms are non-specific. After the onset of neurological symptoms, TBEV RNA is rarely detected in the blood or CSF [74], although persistent viremia has been reported in immunocompromised patients [75]. The duration of viremia is influenced by the environmental and body temperature. In large mammals, viremia is short-lived and only low virus titers are revealed. Birds also pass through a very short viremia stage [76].

## 7. Impact of Climate and Land Use Changes

Tick–host–pathogen interactions are undoubtedly affected by the climate and land use of a region. In general, the development and stability of the TBE natural foci depend on the combination of several ecological factors, including the temperature, relative air humidity, soil moisture, and biotope vegetation characteristics. Warmer and humid conditions favor tick activity and survival. Moreover, the population density and dynamics of ticks and their hosts, the susceptibility of reservoir hosts to TBEV, the proportion of immune hosts, and the virus prevalence among both ticks and vertebrate hosts are also important for the spread of TBEV [36,77,78]. Climate changes may affect the number of ticks through the abundance of their hosts, such as deer, rodents, and birds, and changes to the organic matter environmental compartment [79]. For example, the warming climate in Central Europe is likely to lead to a decline in the density of Norway spruce (*Picea abies*) and the affected areas are likely to be colonized by beech (*Fagus sylvatica*) [80]. The fallen leaves of deciduous trees create a favorable environment for the survival of the free-living stages of ticks.

The results presented by Palo, based on analyses carried out in Sweden, show that climate warming is only a secondary determinant of TBE cases compared to ecological interactions in the host community, such as predator–prey interactions. Low host numbers and a small number of potential host species increase the risk of TBE infections in humans. It has been postulated that TBE transmission is largely determined by interspecies interactions in the tick–host–predator system [81].

Land fragmentation through deforestation and urbanization dividing naturally continuous forests into smaller sections can increase the tick density, because it creates easier movement opportunities for host populations. Another factor that may influence virus transmission is human behavior related to sociological factors—how people spend time outdoors and are exposed to infected ticks [82]. The changes taking place have the overall effect of increasing the spread of arbovirus vectors. For example, an increase in flavivirus cases has been observed in Slovakia. In 2018 and 2019, the molecular screening of selected flaviviruses in vectors (ticks and mosquitoes) revealed the co-circulation of West Nile virus, Usutu virus, and TBEV in that country [83]. 

Mathematical modeling can be used for predictions and the long-term monitoring of tick population dynamics in a given environment. An example is a mathematical model developed to predict the frequency of TBE in the tick–host enzootic cycle. Climate projections were integrated with emerging knowledge on the region-specific enzootic processes of ticks and pathogens by fitting the model parameterization to historical data [84,85].

## 8. TBE and the COVID-19 Pandemic

The impact of COVID-19 on other viral human diseases varies depending on factors such as public health measures, the healthcare infrastructure, and the nature of each specific disease. Respiratory viruses, especially influenza and syncytial viruses, saw a decline in the number of infections during the COVID-19 pandemic, most notably at the beginning of the pandemic [86]. According to data collected by the ECDC, despite the COVID-19 pandemic and the imposed restrictions, the TBE incidence has increased in many countries [3,87]. Ullrich et al. reported an increase in the number of TBE cases in Germany in the first months of the COVID-19 pandemic over the previous years’ total [88]. These studies aimed to assess the impact of the pandemic on the incidence of other notifiable infectious diseases. The prevalence of diseases caused by 32 pathogens was analyzed in the categories of respiratory diseases, gastrointestinal diseases, sexually transmitted diseases, blood-borne diseases, and vector- and healthcare-associated pathogens. The only increase in the number of cases was observed for TBE [88]. Zając et al. analyzed the data published by the ECDC and Eurostat comparing the number of reported TBE and COVID-19 cases in 2020 and TBE cases in 2015–2019 (the reference period). In 12 of the 23 analyzed countries, there was a significant increase in the TBE incidence. The authors concluded that there was no correlation between the incidence of COVID-19 and TBE or between the availability of medical personnel and the TBE incidence in the studied countries [87]. The increase in TBE cases during the COVID-19 pandemic may have been due to a change in people’s behavior and increased outdoor activity, especially in forest areas where there is a higher risk of tick exposure. Perhaps the COVID-19 pandemic period was also associated with less availability of TBE vaccines and a reduction in tick control programs. In addition, less emphasis was placed on preventive healthcare and less attention was paid to other health risks such as vector-borne diseases.

## 9. Vaccination, Treatment, and Prevention

The available protective measure against TBEV infections is immunization. Vaccines against TBEV are based on inactivated whole virions and contain strains of the European or Far-Eastern subtype of TBEV (Table 1). In Europe, FSME IMMUN/TICOVAC and Encepur are produced, based on the European TBEV strains Neudorfl and K23, respectively [89]. In Russia, licensed production takes place of EnceVir, Tick-E-Vac, and its lyophilized analog, TBE vaccine Moscow, based on the Far-Eastern TBEV strains 205 and Sofjin, respectively [90,91]. In China, the SenTaiBao vaccine, based on the Chinese TBEV-FE strain Sen-Zhang, is available [58]. In 2021, the U.S. Food and Drug Administration approved TICOVAC, a tick-borne encephalitis (TBE) vaccine, for use in the United States [92].

Novel TBEV vaccination strategies are in various stages of development (e.g., live attenuated and recombinant types) [93,94]. The new perspectives on TBE vaccine development also extend to mRNA vaccines. In this type of vaccine, mRNA coding for a viral antigen is synthesized in vitro and injected into the host, leading to exogenous protein expression and robust immune responses [95]. It should be mentioned that mRNA-based vaccines have been developed for other flaviviruses, such as the Powassan virus. VanBlargan et al. developed a lipid-nanoparticle-encapsulated modified mRNA vaccine encoding the Powassan virus *prM* and *E* genes. This mRNA vaccine also induced cross-neutralizing antibodies against multiple other tick-borne flaviviruses [96,97,98].

There are no antivirals available against TBEV, although different antiviral compounds are being tested. Several small molecules have been identified that specifically interfere with TBEV replication in vitro, and some of them have also shown therapeutic potential in animal models. For example, nucleoside analogues have been investigated that can inhibit the RNA-dependent RNA polymerase or viral methyltransferases [99]. This group of compounds includes ribavirin, which interferes with TBEV replication and protects infected cells from cytopathic effects in cell culture [100]. Other strategies include human or chimeric monoclonal antibodies with the potential for post-exposure prophylaxis or early therapy [101]. Combination therapy using small-molecule antiviral drugs together with anti-inflammatory agents could both block viral replication processes in host cells and suppress adverse immune responses and cytokine storms [102]. 

Tick control measures rely on the use of ectoparasiticides such as organochlorides, organophosphates, pyrethroids, and, more recently, insect growth regulators and isoxazolines [103]. The most commonly used repellents include N,N-diethyl-meta-toluamide (DEET) and 1-piperidinecarboxylic acid 2-(2-hydroxyethyl)-1-methylpropylester (picaridin) [104]. The appropriate use of chemicals is beneficial in controlling ticks; unfortunately, the misuse, overuse, and inappropriate application of chemical acaricides leads to the development of resistance in the tick population [105]. Acaricides can also be potentially dangerous to human and animal health and can cause food and environmental contamination [106]. Therefore, the development of new agents and effective alternative tick control strategies such as entomopathogenic fungi and plant-based alternatives is required [107,108,109]. Recently, metal, metal oxide, and carbon nanoparticles, particularly those obtained through green fabrication routes, were found to be effective against a wide array of arthropod pests and vectors [110].

An important aspect of protection against TBE is exercising care when in areas with foliage where ticks live. It is recommended to wear long pants, cover the ankles, and avoid walking through tall grass and brush. After traversing a tick habitat, it is advisable to check the skin and take a shower to rinse off any ticks that have not attached. In the case of tick bites with the arachnid attached, the best way to remove it is pulling straight out with tweezers or fingers. The use of grease or ether sprays is not recommended [111].

The TBE surveillance system seems in need of strengthening and extending because its current extent is too limited and the disease’s prevalence is underestimated. A panel of experts published recommendations for improving the TBE surveillance and expanding the vaccine uptake in Europe [112]. The standardization of diagnostic criteria, available tests throughout Europe, and the investigation of all cases of aseptic meningitis/encephalitis of an unknown etiology for possible TBEV infections are suggested. It is also advisable to raise awareness of the risk of TBEV infections and promote vaccination.

## Figures and Tables

**Table 1:** Commercially available inactivated TBE vaccines.

Vaccine	TBEV Strain	Adjuvant	Stabilizer	Distribution
FSME IMMUN/TICOVAC	Neudorfl TBEV-Eu	Al(OH)_3_	HAS (human serum albumin)	Europe, USA
Encepur	K23 TBEV-Eu	Al(OH)_3_	sucrose	Europe
EnceVir	205 TBEV-Fe	Al(OH)_3_	sucrose, HSA	Russian Federation
Tick-E-Vac	Sofjin TBEV-Fe	Al(OH)_3_	sucrose, HSA	Russian Federation
TBE vaccine Moscow	Sofjin TBEV-Fe	Al(OH)_3_	sucrose, HSA, gelatose	Russian Federation
SenTaiBao	Sen-Zhang TBEV-Fe	Al(OH)_3_	HSA	China

## Data Availability

Data sharing is not applicable to this article, no new data were created in this study.

## References

[1] Gritsun T.S., Lashkevich V.A., Gould E.A. (2003). Tick-Borne Encephalitis. Antivir. Res..

[2] Chiffi G., Grandgirard D., Leib S.L., Chrdle A., Růžek D. (2023). Tick-borne Encephalitis: A Comprehensive Review of the Epidemiology, Virology, and Clinical Picture. Rev. Med. Virol..

[3] European Centre for Disease Prevention and Control (2022). Tick-Borne Encephalitis. Annual Epidemiological Report for 2020.

[4] Socha W., Kwasnik M., Larska M., Rola J., Rozek W. (2022). Vector-Borne Viral Diseases as a Current Threat for Human and Animal Health—One Health Perspective. J. Clin. Med..

[5] Ličková M., Fumačová Havlíková S., Sláviková M., Klempa B. (2021). Alimentary Infections by Tick-Borne Encephalitis Virus. Viruses.

[6] Bakhvalova V.N., Panov V.V., Morozova O.V., Ruzek D. (2011). Tick-Borne Encephalitis Virus Quasispecies Rearrangements in Ticks and Mammals. Flavivirus Encephalitis.

[7] Stadler K., Allison S.L., Schalich J., Heinz F.X. (1997). Proteolytic Activation of Tick-Borne Encephalitis Virus by Furin. J. Virol..

[8] Pulkkinen L.I.A., Barrass S.V., Domanska A., Överby A.K., Anastasina M., Butcher S.J. (2022). Molecular Organisation of Tick-Borne Encephalitis Virus. Viruses.

[9] Füzik T., Formanová P., Růžek D., Yoshii K., Niedrig M., Plevka P. (2018). Structure of Tick-Borne Encephalitis Virus and Its Neutralization by a Monoclonal Antibody. Nat. Commun..

[10] Deviatkin A.A., Karganova G.G., Vakulenko Y.A., Lukashev A.N. (2020). TBEV Subtyping in Terms of Genetic Distance. Viruses.

[11] Upstone L., Colley R., Harris M., Goonawardane N. (2023). Functional Characterization of 5′ Untranslated Region (UTR) Secondary RNA Structures in the Replication of Tick-Borne Encephalitis Virus in Mammalian Cells. PLoS Negl. Trop. Dis..

[12] Ng W., Soto-Acosta R., Bradrick S., Garcia-Blanco M., Ooi E. (2017). The 5′ and 3′ Untranslated Regions of the Flaviviral Genome. Viruses.

[13] Morozova O.V., Bakhvalova V.N., Morozov I.V. (2007). Heterogeneity of 3′-Untraslated Region of Genome RNA of the Tick-Borne Encephalitis Virus (TBEV) Strains Isolated from Ticks in the Western Siberia, Russia. Int. J. Biomed. Sci..

[14] Hirano M., Muto M., Sakai M., Kondo H., Kobayashi S., Kariwa H., Yoshii K. (2017). Dendritic Transport of Tick-Borne Flavivirus RNA by Neuronal Granules Affects Development of Neurological Disease. Proc. Natl. Acad. Sci. USA.

[15] Grard G., Moureau G., Charrel R.N., Lemasson J.-J., Gonzalez J.-P., Gallian P., Gritsun T.S., Holmes E.C., Gould E.A., de Lamballerie X. (2007). Genetic Characterization of Tick-Borne Flaviviruses: New Insights into Evolution, Pathogenetic Determinants and Taxonomy. Virology.

[16] Dai X., Shang G., Lu S., Yang J., Xu J. (2018). A New Subtype of Eastern Tick-Borne Encephalitis Virus Discovered in Qinghai-Tibet Plateau, China. Emerg. Microbes Infect..

[17] Kovalev S.Y., Mukhacheva T.A. (2017). Reconsidering the Classification of Tick-Borne Encephalitis Virus within the Siberian Subtype Gives New Insights into Its Evolutionary History. Infect. Genet. Evol..

[18] Kutschera L.S., Wolfinger M.T. (2022). Evolutionary Traits of Tick-Borne Encephalitis Virus: Pervasive Non-Coding RNA Structure Conservation and Molecular Epidemiology. Virus Evol..

[19] Sukhorukov G.A., Paramonov A.I., Lisak O.V., Kozlova I.V., Bazykin G.A., Neverov A.D., Karan L.S. (2023). The Baikal Subtype of Tick-Borne Encephalitis Virus Is Evident of Recombination between Siberian and Far-Eastern Subtypes. PLoS Negl. Trop. Dis..

[20] Kaiser R. (1999). The Clinical and Epidemiological Profile of Tick-Borne Encephalitis in Southern Germany 1994–98. Brain.

[21] Mandl C.W. (2005). Steps of the Tick-Borne Encephalitis Virus Replication Cycle That Affect Neuropathogenesis. Virus Res..

[22] Mansfield K.L., Johnson N., Phipps L.P., Stephenson J.R., Fooks A.R., Solomon T. (2009). Tick-Borne Encephalitis Virus—A Review of an Emerging Zoonosis. J. Gen. Virol..

[23] Charrel R.N., Attoui H., Butenko A.M., Clegg J.C., Deubel V., Frolova T.V., Gould E.A., Gritsun T.S., Heinz F.X., Labuda M. (2004). Tick-Borne Virus Diseases of Human Interest in Europe. Clin. Microbiol. Infect..

[24] Tkachev S.E., Chicherina G.S., Golovljova I., Belokopytova P.S., Tikunov A.Y., Zadora O.V., Glupov V.V., Tikunova N.V. (2017). New Genetic Lineage within the Siberian Subtype of Tick-Borne Encephalitis Virus Found in Western Siberia, Russia. Infect. Genet. Evol..

[25] Kozlova I.V., Verkhozina M.M., Demina T.V., Dzhioev Y.P., Tkachev S.E., Karan L.S., Doroshchenko E.K., Lisak O.V., Suntsova O.V., Paramonov A.I., Tkachev S. (2013). Genetic and Biological Properties of Original TBEV Strains Group Circulating in Eastern Siberia. Encephalitis.

[26] Tkachev S.E., Babkin I.V., Chicherina G.S., Kozlova I.V., Verkhozina M.M., Demina T.V., Lisak O.V., Doroshchenko E.K., Dzhioev Y.P., Suntsova O.V. (2020). Genetic Diversity and Geographical Distribution of the Siberian Subtype of the Tick-Borne Encephalitis Virus. Ticks Tick-Borne Dis..

[27] Mandl C.W., Heinz F.X., Holzmann H., Kunz C., Ecker M. (1997). Infectious CDNA Clones of Tick-Borne Encephalitis Virus European Subtype Prototypic Strain Neudoerfl and High Virulence Strain Hypr. J. Gen. Virol..

[28] Dumpis U., Crook D., Oksi J. (1999). Tick-Borne Encephalitis. Clin. Infect. Dis..

[29] Chitimia-Dobler L., Lemhöfer G., Król N., Bestehorn M., Dobler G., Pfeffer M. (2019). Repeated Isolation of Tick-Borne Encephalitis Virus from Adult Dermacentor Reticulatus Ticks in an Endemic Area in Germany. Parasites Vectors.

[30] Krivanec K., Kopecký J., Tomková E., Grubhoffer L. (1988). Isolation of TBE Virus from the Tick Ixodes Hexagonus. Folia Parasitol..

[31] Lichard M., Kozuch O. (1967). Persistence of Tick-Borne Encephalitis Virus in Nymphs and Adults of Ixodes Arboricola and Its Transmission to White Mice. Acta Virol..

[32] Hubálek Z., Rudolf I. (2012). Tick-Borne Viruses in Europe. Parasitol. Res..

[33] Riedl H., Kozuch O., Sixl W., Schmeller E., Nosek J. (1971). Isolation of the tick-borne encephalitis virus (TBE-virus) from the tick Haemaphysalis concinna Koch. Arch. Hyg. Bakteriol..

[34] Kozuch O., Nosek J. (1971). Transmission of Tick-Borne Encephalitis (TBE) Virus by Dermacentor Marginatus and D. Reticulatus Ticks. Acta Virol..

[35] Nosek J., Ciampor F., Kozuch O., Rajcáni J. (1972). Localization of Tick-Borne Encephalitis Virus in Alveolar Cells of Salivary Glands of Dermacentor Marginatus and Haemaphysalis Inermis Ticks. Acta Virol..

[36] Bakhvalova V.N., Dobrotvorsky A.K., Panov V.V., Matveeva V.A., Tkachev S.E., Morozova O.V. (2006). Natural Tick-Borne Encephalitis Virus Infection among Wild Small Mammals in the Southeastern Part of Western Siberia, Russia. Vector Borne Zoonotic Dis..

[37] Bakhvalova V.N., Potapova O.F., Panov V.V., Morozova O.V. (2009). Vertical Transmission of Tick-Borne Encephalitis Virus between Generations of Adapted Reservoir Small Rodents. Virus Res..

[38] Wilhelmsson P., Jaenson T.G.T., Olsen B., Waldenström J., Lindgren P.-E. (2020). Migratory Birds as Disseminators of Ticks and the Tick-Borne Pathogens Borrelia Bacteria and Tick-Borne Encephalitis (TBE) Virus: A Seasonal Study at Ottenby Bird Observatory in South-Eastern Sweden. Parasites Vectors.

[39] Michelitsch A., Wernike K., Klaus C., Dobler G., Beer M. (2019). Exploring the Reservoir Hosts of Tick-Borne Encephalitis Virus. Viruses.

[40] Holding M., Dowall S.D., Medlock J.M., Carter D.P., McGinley L., Curran-French M., Pullan S.T., Chamberlain J., Hansford K.M., Baylis M. (2019). Detection of New Endemic Focus of Tick-Borne Encephalitis Virus (TBEV), Hampshire/Dorset Border, England, September 2019. Euro Surveill..

[41] Jääskeläinen A., Tonteri E., Pieninkeroinen I., Sironen T., Voutilainen L., Kuusi M., Vaheri A., Vapalahti O. (2016). Siberian Subtype Tick-Borne Encephalitis Virus in Ixodes Ricinus in a Newly Emerged Focus, Finland. Ticks Tick Borne Dis..

[42] Suzuki Y. (2007). Multiple Transmissions of Tick-Borne Encephalitis Virus between Japan and Russia. Genes. Genet. Syst..

[43] Cerný V. (1975). The Role of Mammals in Natural Foci of Tick-Borne Encephalitis in Central Europe. Folia Parasitol..

[44] Klaus C., Gethmann J., Hoffmann B., Ziegler U., Heller M., Beer M. (2016). Tick Infestation in Birds and Prevalence of Pathogens in Ticks Collected from Different Places in Germany. Parasitol. Res..

[45] Hofmeester T.R., Sprong H., Jansen P.A., Prins H.H.T., van Wieren S.E. (2017). Deer Presence Rather than Abundance Determines the Population Density of the Sheep Tick, Ixodes Ricinus, in Dutch Forests. Parasit. Vectors.

[46] Krzysiak M.K., Anusz K., Konieczny A., Rola J., Salat J., Strakova P., Olech W., Larska M. (2021). The European Bison (Bison Bonasus) as an Indicatory Species for the Circulation of Tick-Borne Encephalitis Virus (TBEV) in Natural Foci in Poland. Ticks Tick-Borne Dis..

[47] Salat J., Ruzek D. (2020). Tick-Borne Encephalitis in Domestic Animals. Acta Virol..

[48] Böhm B., Schade B., Bauer B., Hoffmann B., Hoffmann D., Ziegler U., Beer M., Klaus C., Weissenböck H., Böttcher J. (2017). Tick-Borne Encephalitis in a Naturally Infected Sheep. BMC Vet. Res..

[49] Holzmann H., Aberle S.W., Stiasny K., Werner P., Mischak A., Zainer B., Netzer M., Koppi S., Bechter E., Heinz F.X. (2009). Tick-Borne Encephalitis from Eating Goat Cheese in a Mountain Region of Austria. Emerg. Infect. Dis..

[50] Buczek A.M., Buczek W., Buczek A., Wysokińska-Miszczuk J. (2022). Food-Borne Transmission of Tick-Borne Encephalitis Virus-Spread, Consequences, and Prophylaxis. Int. J. Environ. Res. Public Health.

[51] Levkovich E.N., Pogodina V.V. (1958). Infection through the alimentary tract with tick-borne encephalitis. Vopr. Virusol..

[52] Bogovic P., Strle F. (2015). Tick-Borne Encephalitis: A Review of Epidemiology, Clinical Characteristics, and Management. World J. Clin. Cases.

[53] Avšič-Županc T., Poljak M., Matičič M., Radšel-Medvešček A., LeDuc J.W., Stiasny K., Kunz C., Heinz F.X. (1995). Laboratory Acquired Tick-Borne Meningoencephalitis: Characterisation of Virus Strains. Clin. Diagn. Virol..

[54] Wahlberg P., Saikku P., Brummer-Korvenkontio M. (1989). Tick-Borne Viral Encephalitis in Finland. The Clinical Features of Kumlinge Disease during 1959-1987. J. Intern. Med..

[55] Lipowski D., Popiel M., Perlejewski K., Nakamura S., Bukowska-Ośko I., Rzadkiewicz E., Dzieciątkowski T., Milecka A., Wenski W., Ciszek M. (2017). A Cluster of Fatal Tick-Borne Encephalitis Virus Infection in Organ Transplant Setting. J. Infect. Dis..

[56] Kerlik J., Avdičová M., Musilová M., Bérešová J., Mezencev R. (2022). Breast Milk as Route of Tick-Borne Encephalitis Virus Transmission from Mother to Infant. Emerg. Infect. Dis..

[57] Beauté J., Spiteri G., Warns-Petit E., Zeller H. (2018). Tick-Borne Encephalitis in Europe, 2012 to 2016. Euro Surveill..

[58] Yoshii K., Song J.Y., Park S.-B., Yang J., Schmitt H.-J. (2017). Tick-Borne Encephalitis in Japan, Republic of Korea and China. Emerg. Microbes Infect..

[59] Chen X., Li F., Yin Q., Liu W., Fu S., He Y., Lei W., Xu S., Liang G., Wang S. (2019). Epidemiology of Tick-Borne Encephalitis in China, 2007–2018. PLoS ONE.

[60] Uchida L., Hayasaka D., Ngwe Tun M.M., Morita K., Muramatsu Y., Hagiwara K. (2018). Survey of Tick-Borne Zoonotic Viruses in Wild Deer in Hokkaido, Japan. J. Vet. Med. Sci..

[61] Yoshii K., Takahashi-Iwata I., Shirai S., Kobayashi S., Yabe I., Sasaki H. (2020). A Retrospective Epidemiological Study of Tick-Borne Encephalitis Virus in Patients with Neurological Disorders in Hokkaido, Japan. Microorganisms.

[62] Kim S.-Y., Yun S.-M., Han M.G., Lee I.Y., Lee N.Y., Jeong Y.E., Lee B.C., Ju Y.R. (2008). Isolation of Tick-Borne Encephalitis Viruses from Wild Rodents, South Korea. Vector-Borne Zoonotic Dis..

[63] Holding M., Dowall S.D., Medlock J.M., Carter D.P., Pullan S.T., Lewis J., Vipond R., Rocchi M.S., Baylis M., Hewson R. (2020). Tick-Borne Encephalitis Virus, United Kingdom. Emerg. Infect. Dis..

[64] Jahfari S., de Vries A., Rijks J.M., Van Gucht S., Vennema H., Sprong H., Rockx B. (2017). Tick-Borne Encephalitis Virus in Ticks and Roe Deer, the Netherlands. Emerg. Infect. Dis..

[65] Ternovoi V.A., Kurzhukov G.P., Sokolov Y.V., Ivanov G.Y., Ivanisenko V.A., Loktev A.V., Ryder R.W., Netesov S.V., Loktev V.B. (2003). Tick-Borne Encephalitis with Hemorrhagic Syndrome, Novosibirsk Region, Russia, 1999. Emerg. Infect. Dis..

[66] Bogovic P., Lotric-Furlan S., Strle F. (2010). What Tick-Borne Encephalitis May Look like: Clinical Signs and Symptoms. Travel Med. Infect. Dis..

[67] Ergunay K., Tkachev S., Kozlova I., Růžek D. (2016). A Review of Methods for Detecting Tick-Borne Encephalitis Virus Infection in Tick, Animal, and Human Specimens. Vector Borne Zoonotic Dis..

[68] Taba P., Schmutzhard E., Forsberg P., Lutsar I., Ljøstad U., Mygland Å., Levchenko I., Strle F., Steiner I. (2017). EAN Consensus Review on Prevention, Diagnosis and Management of Tick-Borne Encephalitis. Eur. J. Neurol..

[69] Holzmann H. (2003). Diagnosis of Tick-Borne Encephalitis. Vaccine.

[70] Niedrig M., Vaisviliene D., Teichmann A., Klockmann U., Biel S.S. (2001). Comparison of Six Different Commercial IgG-ELISA Kits for the Detection of TBEV-Antibodies. J. Clin. Virol..

[71] Günther G., Haglund M., Lindquist L., Sköldenberg B., Forsgren M. (1997). Intrathecal IgM, IgA and IgG Antibody Response in Tick-Borne Encephalitis. Long-Term Follow-up Related to Clinical Course and Outcome. Clin. Diagn. Virol..

[72] Reusken C., Boonstra M., Rugebregt S., Scherbeijn S., Chandler F., Avšič-Županc T., Vapalahti O., Koopmans M., GeurtsvanKessel C.H. (2019). An Evaluation of Serological Methods to Diagnose Tick-Borne Encephalitis from Serum and Cerebrospinal Fluid. J. Clin. Virol..

[73] Donoso Mantke O., Aberle S.W., Avšič-Županc T., Labuda M., Niedrig M. (2007). Quality Control Assessment for the PCR Diagnosis of Tick-Borne Encephalitis Virus Infections. J. Clin. Virol..

[74] Saksida A., Duh D., Lotric-Furlan S., Strle F., Petrovec M., Avsic-Zupanc T. (2005). The Importance of Tick-Borne Encephalitis Virus RNA Detection for Early Differential Diagnosis of Tick-Borne Encephalitis. J. Clin. Virol..

[75] Caracciolo I., Bassetti M., Paladini G., Luzzati R., Santon D., Merelli M., Sabbata G.D., Carletti T., Marcello A., D’Agaro P. (2015). Persistent Viremia and Urine Shedding of Tick-Borne Encephalitis Virus in an Infected Immunosuppressed Patient from a New Epidemic Cluster in North-Eastern Italy. J. Clin. Virol..

[76] Pustijanac E., Buršić M., Talapko J., Škrlec I., Meštrović T., Lišnjić D. (2023). Tick-Borne Encephalitis Virus: A Comprehensive Review of Transmission, Pathogenesis, Epidemiology, Clinical Manifestations, Diagnosis, and Prevention. Microorganisms.

[77] Pukhovskaya N.M., Morozova O.V., Vysochina N.P., Belozerova N.B., Bakhmetyeva S.V., Zdanovskaya N.I., Seligman S.J., Ivanov L.I. (2018). Tick-Borne Encephalitis Virus in Arthropod Vectors in the Far East of Russia. Ticks Tick. Borne Dis..

[78] Bakhvalova V.N., Chicherina G.S., Potapova O.F., Panov V.V., Glupov V.V., Potapov M.A., Seligman S.J., Morozova O.V. (2016). Tick-Borne Encephalitis Virus Diversity in Ixodid Ticks and Small Mammals in South-Western Siberia, Russia. Vector Borne Zoonotic Dis..

[79] Gray J.S., Dautel H., Estrada-Peña A., Kahl O., Lindgren E. (2009). Effects of Climate Change on Ticks and Tick-Borne Diseases in Europe. Interdiscip. Perspect. Infect. Dis..

[80] Heine P., Hausen J., Ottermanns R., Schäffer A., Roß-Nickoll M. (2019). Forest Conversion from Norway Spruce to European Beech Increases Species Richness and Functional Structure of Aboveground Macrofungal Communities. For. Ecol. Manag..

[81] Palo R.T. (2014). Tick-Borne Encephalitis Transmission Risk: Its Dependence on Host Population Dynamics and Climate Effects. Vector-Borne Zoonotic Dis..

[82] Randolph S.E., On Behalf of The Eden-Tbd Sub-Project Team C. (2010). Human Activities Predominate in Determining Changing Incidence of Tick-Borne Encephalitis in Europe. Eurosurveillance.

[83] Čabanová V., Kerlik J., Kirschner P., Rosochová J., Klempa B., Sláviková M., Ličková M. (2023). Co-Circulation of West Nile, Usutu, and Tick-Borne Encephalitis Viruses in the Same Area: A Great Challenge for Diagnostic and Blood and Organ Safety. Viruses.

[84] Tardy O., Acheson E.S., Bouchard C., Chamberland É., Fortin A., Ogden N.H., Leighton P.A. (2023). Mechanistic Movement Models to Predict Geographic Range Expansions of Ticks and Tick-Borne Pathogens: Case Studies with Ixodes Scapularis and Amblyomma Americanum in Eastern North America. Ticks Tick-Borne Dis..

[85] Nah K., Bede-Fazekas Á., Trájer A.J., Wu J. (2020). The Potential Impact of Climate Change on the Transmission Risk of Tick-Borne Encephalitis in Hungary. BMC Infect. Dis..

[86] Chow E.J., Uyeki T.M., Chu H.Y. (2022). The Effects of the COVID-19 Pandemic on Community Respiratory Virus Activity. Nat. Rev. Microbiol..

[87] Zając Z., Bartosik K., Kulisz J., Woźniak A. (2022). Incidence of Tick-Borne Encephalitis during the COVID-19 Pandemic in Selected European Countries. J. Clin. Med..

[88] Ullrich A., Schranz M., Rexroth U., Hamouda O., Schaade L., Diercke M., Boender T.S. (2021). Impact of the COVID-19 Pandemic and Associated Non-Pharmaceutical Interventions on Other Notifiable Infectious Diseases in Germany: An Analysis of National Surveillance Data during Week 1–2016—Week 32–2020. Lancet Reg. Health Eur..

[89] Kollaritsch H., Paulke-Korinek M., Holzmann H., Hombach J., Bjorvatn B., Barrett A. (2012). Vaccines and Vaccination against Tick-Borne Encephalitis. Expert Rev. Vaccines.

[90] Šmit R., Postma M.J. (2015). Review of Tick-Borne Encephalitis and Vaccines: Clinical and Economical Aspects. Expert Rev. Vaccines.

[91] Tuchynskaya K., Volok V., Illarionova V., Okhezin E., Polienko A., Belova O., Rogova A., Chernokhaeva L., Karganova G. (2021). Experimental Assessment of Possible Factors Associated with Tick-Borne Encephalitis Vaccine Failure. Microorganisms.

[92] Hills S.L., Broussard K.R., Broyhill J.C., Shastry L.G., Cossaboom C.M., White J.L., Machesky K.D., Kosoy O., Girone K., Klena J.D. (2022). Tick-Borne Encephalitis among US Travellers, 2010–2020. J. Travel Med..

[93] Rumyantsev A.A., Chanock R.M., Murphy B.R., Pletnev A.G. (2006). Comparison of Live and Inactivated Tick-Borne Encephalitis Virus Vaccines for Safety, Immunogenicity and Efficacy in Rhesus Monkeys. Vaccine.

[94] Ershova A.S., Gra O.A., Lyaschuk A.M., Grunina T.M., Tkachuk A.P., Bartov M.S., Savina D.M., Sergienko O.V., Galushkina Z.M., Gudov V.P. (2016). Recombinant Domains III of Tick-Borne Encephalitis Virus Envelope Protein in Combination with Dextran and CpGs Induce Immune Response and Partial Protectiveness against TBE Virus Infection in Mice. BMC Infect. Dis..

[95] Wollner C.J., Richner J.M. (2021). MRNA Vaccines against Flaviviruses. Vaccines.

[96] VanBlargan L.A., Himansu S., Foreman B.M., Ebel G.D., Pierson T.C., Diamond M.S. (2018). An MRNA Vaccine Protects Mice against Multiple Tick-Transmitted Flavivirus Infections. Cell Rep..

[97] Mandl C.W., Aberle J.H., Aberle S.W., Holzmann H., Allison S.L., Heinz F.X. (1998). In Vitro-Synthesized Infectious RNA as an Attenuated Live Vaccine in a Flavivirus Model. Nat. Med..

[98] Kofler R.M., Aberle J.H., Aberle S.W., Allison S.L., Heinz F.X., Mandl C.W. (2004). Mimicking Live Flavivirus Immunization with a Noninfectious RNA Vaccine. Proc. Natl. Acad. Sci. USA.

[99] Eyer L., Nencka R., de Clercq E., Seley-Radtke K., Růžek D. (2018). Nucleoside Analogs as a Rich Source of Antiviral Agents Active against Arthropod-Borne Flaviviruses. Antivir. Chem. Chemother..

[100] Tang W.-D., Tang H.-L., Peng H.-R., Ren R.-W., Zhao P., Zhao L.-J. (2023). Inhibition of Tick-Borne Encephalitis Virus in Cell Cultures by Ribavirin. Front. Microbiol..

[101] VanBlargan L.A., Errico J.M., Kafai N.M., Burgomaster K.E., Jethva P.N., Broeckel R.M., Meade-White K., Nelson C.A., Himansu S., Wang D. (2021). Broadly Neutralizing Monoclonal Antibodies Protect against Multiple Tick-Borne Flaviviruses. J. Exp. Med..

[102] Eyer L., Seley-Radtke K., Ruzek D. (2023). New Directions in the Experimental Therapy of Tick-Borne Encephalitis. Antivir. Res..

[103] Wardhaugh K.G. (2005). Insecticidal Activity of Synthetic Pyrethroids, Organophosphates, Insect Growth Regulators, and Other Livestock Parasiticides: An Australian Perspective. Environ. Toxicol. Chem..

[104] Antwi F.B., Shama L.M., Peterson R.K.D. (2008). Risk Assessments for the Insect Repellents DEET and Picaridin. Regul. Toxicol. Pharmacol..

[105] George J.E., Pound J.M., Davey R.B. (2004). Chemical Control of Ticks on Cattle and the Resistance of These Parasites to Acaricides. Parasitology.

[106] Van Wieren S.E., Braks M.A.H., Lahr J., Braks M.A.H., Van Wieren S.E., Takken W., Sprong H. (2016). 19. Effectiveness and Environmental Hazards of Acaricides Applied to Large Mammals for Tick Control. Ecology and Control of Vector-borne Diseases.

[107] Banumathi B., Vaseeharan B., Rajasekar P., Prabhu N.M., Ramasamy P., Murugan K., Canale A., Benelli G. (2017). Exploitation of Chemical, Herbal and Nanoformulated Acaricides to Control the Cattle Tick, Rhipicephalus (Boophilus) Microplus—A Review. Vet. Parasitol..

[108] Nana P., Ekesi S., Nchu F., Maniania N.K. (2016). Compatibility of *Metarhizium anisopliae* with *Calpurnia aurea* Leaf Extracts and Virulence against *Rhipicephalus pulchellus*. J. Appl. Entomol..

[109] Adenubi O.T., Ahmed A.S., Fasina F.O., McGaw L.J., Eloff J.N., Naidoo V. (2018). Pesticidal Plants as a Possible Alternative to Synthetic Acaricides in Tick Control: A Systematic Review and Meta-Analysis. Ind. Crops Prod..

[110] Benelli G., Maggi F., Romano D., Stefanini C., Vaseeharan B., Kumar S., Higuchi A., Alarfaj A.A., Mehlhorn H., Canale A. (2017). Nanoparticles as Effective Acaricides against Ticks—A Review. Ticks Tick-Borne Dis..

[111] Rahlenbeck S., Fingerle V., Doggett S. (2016). Prevention of Tick-Borne Diseases: An Overview. Br. J. Gen. Pract..

[112] Kunze M., Banović P., Bogovič P., Briciu V., Čivljak R., Dobler G., Hristea A., Kerlik J., Kuivanen S., Kynčl J. (2022). Recommendations to Improve Tick-Borne Encephalitis Surveillance and Vaccine Uptake in Europe. Microorganisms.

